# Diagnostic accuracy of urine HE4 in patients with ovarian cancer: a meta-analysis

**DOI:** 10.18632/oncotarget.14173

**Published:** 2016-12-26

**Authors:** Meng-Meng Jia, Jie Deng, Xiao-lin Cheng, Zhen Yan, Qing-Chun Li, Ying-Ying Xing, Dong-Mei Fan, Xiao-Yu Tian

**Affiliations:** ^1^ Department of Obstetrics and Gynecology, The First Affiliated Hospital, and College of Clinical Medicine of Henan University of Science and Technology, Luoyang 471003, China

**Keywords:** HE4, tumour marker, ovarian cancer, diagnosis, meta-analysis

## Abstract

Urine HE4 has been reported as the potential novel diagnostic biomarker for ovarian cancer in several studies, but their results were inconsistent. Therefore, we conducted a systematic analysis to evaluate the diagnostic value of urine HE4 in detecting ovarian cancer. A comprehensive electronic and manual search was conducted for relevant literatures through several databases up to May 5, 2016. The quality of the studies included in the systematic review was assessed using the Quality Assessment of Diagnostic Accuracy Studies (QUADAS-2) tool. All analyses were conducted using Meta-DiSc 1.4 and STATA 12.0 software. A total of seven publications were included in this study, and these studies included 413 ovarian cancer patients and 573 controls. The summary estimates were: sensitivity 0.76 (95% confidence interval [CI]: 0.72–0.80), specificity 0.92 (95% CI: 0.89–0.94), positive likelihood ratio 8.39 (95%CI: 4.81–14.63), negative likelihood ratio 0.23 (95% CI: 0.13–0.39), diagnostic odds ratio 37.90 (95% CI: 18.69–76.83), and area under the curve 0.93. According to our results, urine HE4 has greater diagnostic value in detecting ovarian cancer. In addition, considering the high heterogeneity, further research studies with more well-designed and large sample sizes are needed in the future.

## INTRODUCTION

Ovarian cancer, the most lethal tumour type among gynaecologic cancers, is the fifth most common cause of cancer death in women [1]. Each year, 220 000 women develop epithelial ovarian cancer worldwide [2]. Since its lethality may be due to the lack of specific symptoms, effective screening strategies and early diagnostic methods, over 75% of the patients are diagnosed at an advanced stage (stage III or IV), and the 5-year survival rate is only 20%-25% [3]. Nevertheless, the 5-year survival rate for stage I ovarian cancer is up to 90%. Therefore, the early diagnosis of malignant ovarian tumours is a key factor for improving the survival rate of patients.

Histopathological examination is currently considered the gold standard in the diagnosis of ovarian cancer, but the invasive nature of obtaining tumour samples creates a challenge that limits its application in the early diagnosis of ovarian cancer because most patients have no symptoms. Thus, bimanual pelvic examination, transvaginal sonography (TVS) and carbohydrate antigen (CA) 125 levels are widely employed diagnostic tools for the early detection of ovarian cancer. Unfortunately, several high-quality studies have demonstrated that bimanual pelvic examination lacks accuracy as a screening method for distinguishing benign from malignant lesions [4]. TVS is accurate in detecting abnormalities in ovarian volume and morphology but is less reliable in differentiating benign from malignant ovarian tumours, and its diagnostic accuracy is largely affected by the experience of the examiner [5]. CA125 was the only FDA-approved biomarker for ovarian cancer before 2008. Although CA125 is elevated in 80% of women with advanced ovarian cancer, it is increased in only 50% of women with early stage ovarian cancer [6]. Furthermore, CA125 levels are elevated in some benign gynaecological diseases (including endometriosis) and non-gynaecological malignancies [7]. Therefore, it may be necessary to identify other biomarkers for the early detection and disease surveillance of ovarian cancer.

Among a wide spectrum of biomarkers, human epidermis protein 4 (HE4) is the most promising. HE4 protein is encoded by WAP four-disulfide core domain 2 (WFDC2) [8], which was found to be highly expressed in ovarian carcinoma, especially in serous and endometrioid cancers [9]. Unlike CA125, HE4 is not overexpressed in benign ovarian disease, normal ovarian tissue or tumours with low malignant potential [10]. In 2008, HE4 was the first biomarker since CA125 to be approved by the FDA for monitoring patients with ovarian cancer for disease recurrence. In 2010, Hellstrom I et al reported higher urinary concentrations of HE4 in patients with early and late stage ovarian cancer, indicating that urinary and serum HE4 measurements had similar sensitivity and specificity [11]. Although extensive analyses have indicated the potential of urine HE4 as a novel diagnostic marker for ovarian cancer, the previous studies have been limited by relatively small patient populations, and no previously published meta-analyses have addressed this research question. In the present study, we conducted a meta-analysis of data from multiple studies to systematically evaluate the potential of urine HE4 as a non-invasive biomarker for the diagnosis of ovarian cancer.

## RESULTS

### Screening results

**Figure 1:**
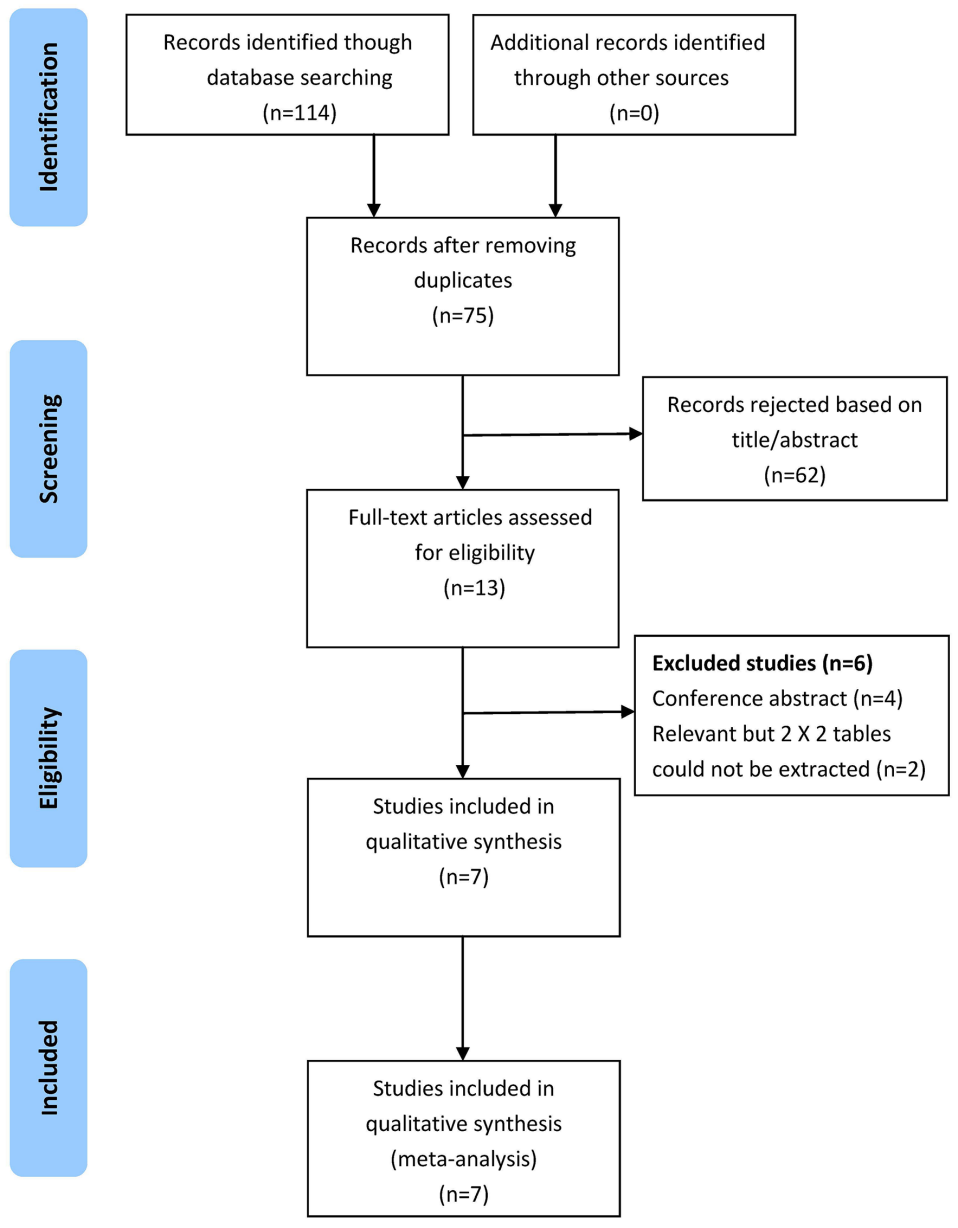
Flowchart depicting the study selection process for this systematic review and meta-analysis

As show in Figure 1, 114 potentially relevant articles were retrieved after the initial database searches. After removing duplicates, we obtained 75 publications. Of these 75 articles, we excluded 62 that were not relevant to our study on the basis of title and abstract. Afterward, the remaining 13 articles were subjected to a full-text review, and 6 articles were excluded (four conference papers and two articles without sufficient information to calculate the sensitivity and specificity). Consequently, we obtained 7 publications [11–17] that met all of the inclusion and exclusion criteria for this meta-analysis.

### Characteristics of the included studies and quality assessments

In this meta-analysis, the final set of 7 diagnostic studies included a total of 413 ovarian cancer patients and 573 controls (patients with benign gynaecological disease and healthy women). All the ovarian cancer patients were diagnosed based on histopathological examination. Regarding the origin of the studies, four studies were performed in Asia (China) [13–16], which compared the value of serum HE4 and urine HE4 in the diagnosis of ovarian cancer, and three were conducted in European countries [11, 12, 17], which analyzed the value of urine HE4 in the diagnosis of ovarian cancer. All the studies were published from 2010 to 2015, and the sample size ranged from 78 to 279. Enzyme-linked immunosorbent assay (ELISA) was used in these studies to determine urine HE4 and serum HE4 levels. The characteristics of the included studies are shown in Table 1.

**Table 1:** Summary of the diagnostic results of the included studies

study	Year	Country	Simple size		Simple type		Test methods	TP (n)	FP (n)	FN (n)	TN (n)	Cut-off Values (pmol/L)
			Cases	Controls				a	b	c	d	
Hellstrom	2010	USA	79	56	Urine	/	ELISA	70	5	9	51	NR
Macuks	2012	Riga	23	55	Urine	/	ELISA	18	14	5	41	1300
Jian HQ	2012	China	30	78	Urine	Serum	ELISA	28	1	2	77	71.519
Yuan ZF	2012	China	50	78	Urine	Serum	ELISA	40	7	10	71	6.51
Zhang YJ	2013	China	98	57	Urine	Serum	ELISA	84	7	14	50	18.74
Wang Y	2014	China	41	62	Urine	Serum	ELISA	28	4	13	58	6.51
Liao	2015	USA	92	187	Urine	/	ELISA	47	10	45	177	Specificity of 95%

Abbreviations: ELASA, Enzyme Linked Immunosorbent Assay; TP, true positive rate; FP, false-positive rate; FN, false-negative rate; TN, true negative rate; NR, not reported;

**Figure 2:**
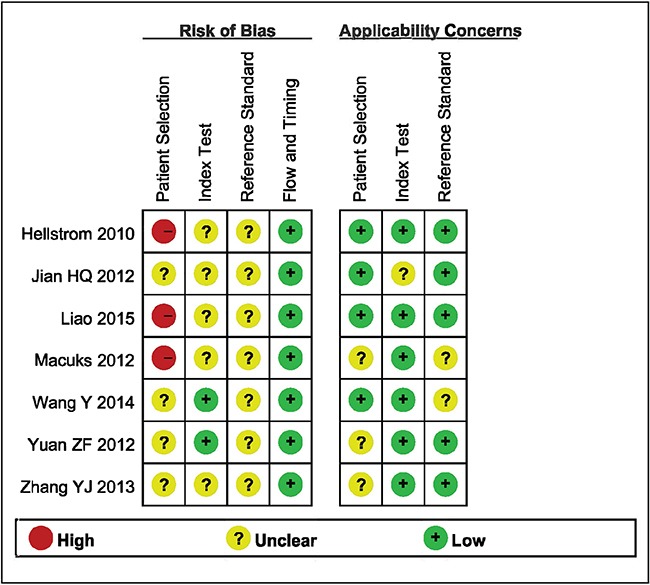
Summary the assessment of methodological quality of included studies by QUADAS-2 tool

Details of the methodological assessment are shown in Figure 2. We evaluated the quality of the seven included studies using the QUADAS-2 tool. According to the results of the methodological assessment, all the included studies were of acceptable quality.

### Diagnostic accuracy

**Figure 3:**
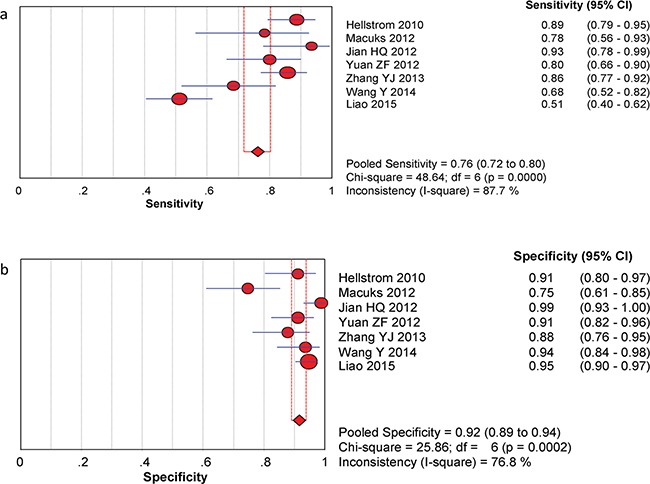
Forest plots of estimated sensitivity (a) and specifcity (b) for urine HE4 in the diagnosis of ovarian cancer **a**. Forest plots of estimated sensitivity. **b**. Forest plots of estimated specifcity.

**Figure 4:**
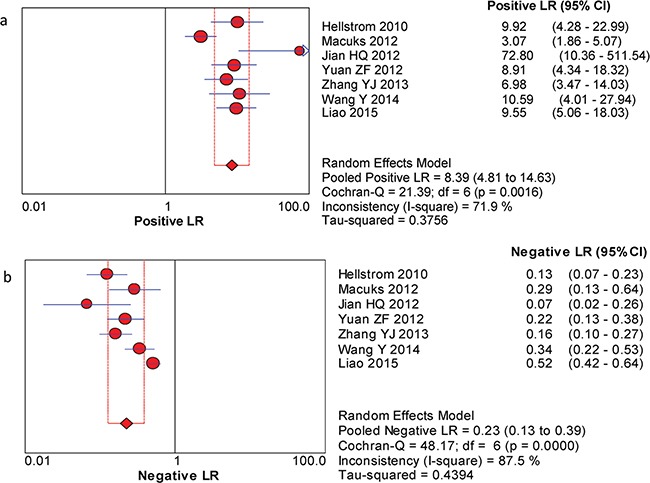
Forest plots of estimated PLR (a) and NLR (b) for urine HE4 in the diagnosis of ovarian cancer **a**. Forest plots of estimated positive LR. **b**. Forest plots of estimated negative LR.

**Figure 5:**
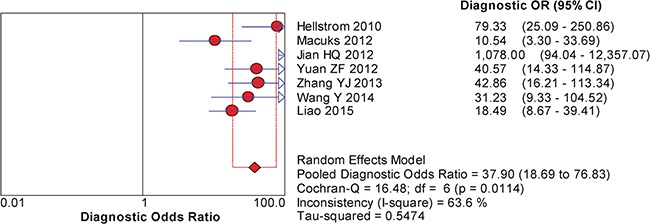
Forest plots of the pooled diagnostic odds ratio (DOR) for urine HE4 in the diagnosis of ovarian cancer

**Figure 6:**
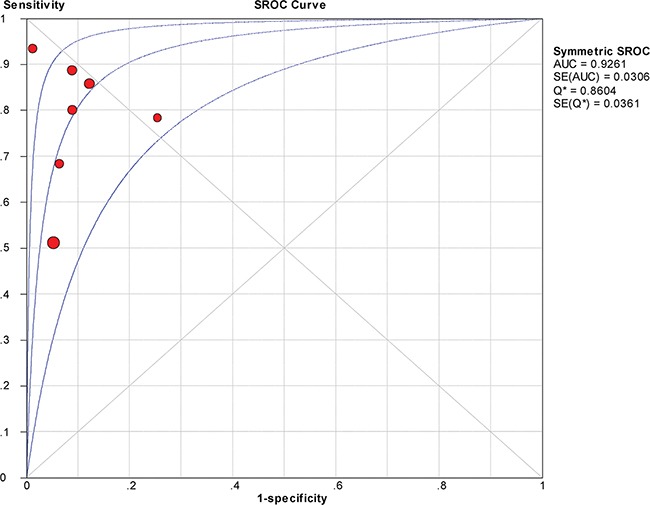
Summary receiver operating characteristic (SROC) curve for urine HE4 in the diagnosis of ovarian cancer

The between-study variability (i.e., heterogeneity) was high for both sensitivity (I^2^ = 87.7%) and specificity (I^2^ = 76.8%), suggesting high levels of heterogeneity in the seven studies. Therefore, the random effects model was applied. The threshold effect was the major cause of heterogeneity. In the meta-analysis, the Spearman correlation coefficient was -0.071 (P = 0.879), confirming that the threshold effect was not significant and that the heterogeneity was caused by other factors. Overall, the pooled sensitivity and specificity were 0.76 (95% CI, 0.72–0.80) (Figure 3a) and 0.92 (95% CI, 0.89–0.94) (Figure 3b), respectively. In addition, the pooled PLR was 8.39 (95% CI, 4.81–14.63) (Figure 4a), the NLR was 0.23 (95% CI, 0.13–0.39) (Figure 4b), and the DOR was 37.90 (95% CI, 18.69–76.83) (Figure 5). The AUC of the SROC was 0.93 (standard error (SE) = 0.03) with a Q* of 0.86 (SE = 0.04) (Figure 6). These results demonstrated that urine HE4 is an effective diagnostic marker for ovarian cancer.

Besides, the pooled SEN of serum HE4 in the 4 Asian studies for the diagnosis of ovarian cancer was 0.74 (95% CI, 0.68–0.80) and the pooled SPE was 0.88 (95% CI, 0.84–0.92). The PLR was 5.69 (95% CI, 3.60–9.00), the NLR was 0.30 (95% CI, 0.17–0.55), and the DOR was 19.39 (95% CI, 7.11–52.90), respectively. The AUC of the SROC was 0.95 (Table 2).

**Table 2:** The result of subgroup analyses and sensitivity analysis

Variables	SEN (95% CI)	I^2^	SPE (95% CI)	I^2^	PLR (95% CI)	NLR (95% CI)	DOR (95% CI)	AUC
**Subgroup analysis**								
Asia	0.82 (0.72-0.80)	66.1	0.93 (0.89-0.96)	63.7	10.22 (5.54-18.85)	0.20 (0.12-0.34)	56.40 (21.54- 147.68)	0.92
European	0.70 (0.63-0.76)	93.5	0.90 (0.86-0.93)	87.6	6.42 (2.56-16.10)	0.27 (0.10-0.74)	24.37 (8.48- 69.99)	0.91
**Sample types**								
Serum	0.74 (0.68-0.80)	85.0	0.88 (0.84-0.92)	0.0	5.69 (3.60-9.00)	0.30 (0.17-0.55)	19.39 (7.11- 52.90)	0.95
**Sensitivity analysis**								
Hellstrom 2010	0.73 (0.68-0.78)	87.3	0.92 (0.89-0.94)	80.6	8.29 (4.38-15.71)	0.25 (0.15-0.44)	33.40 (15.56- 71.72)	0.91
Macuks 2012	0.76 (0.72-0.80)	89.7	0.93 (0.91-0.95)	46.1	9.49 (6.79-13.28)	0.21 (0.12-0.40)	47.17 (22.35- 95.38)	0.95
Jian HQ 2012	0.75 (0.70-0.79)	88.1	0.91 (0.88-0.93)	71.2	7.22 (4.45-11.71)	0.25 (0.14-0.43)	46.17 (22.35- 53.62)	0.92
Yuan ZF 2012	0.76 (0.71-0.80)	89.6	0.92 (0.89-0.94)	80.6	8.52 (4.38-16.60)	0.22 (0.12-0.42)	38.66 (16,59- 90.06)	0.93
Zhang YJ 2013	0.73 (0.68-0.78)	88.0	0.92 (0.89-0.94)	79.8	8.99 (4.52-17.88)	0.24 (0.13-0.43)	38.29 (16.33- 89.78)	0.92
Wang Y 2014	0.77 (0.73-0.81)	89.4	0.91 (0.89-0.94)	80.4	8.21 (4.39-15.37)	0.20 (0.10-0.41)	40.24 (17.56-92.23)	0.93
Liao 2015	0.83 (0.79-0.87)	55.3	0.90 (0.87-0.93)	77.6	8.42 (4.29-16.53)	0.20 (0.13-0.29)	45.30 (19.89- 103.20)	0.91
Total	0.76 (0.72-0.80)	87.7	0.92 (0.89-0.94)	76.8	8.39 (4.81-14.63)	0.23 (0.13-0.39)	37.90 (18.69-76.83)	0.93

Abbreviations: SEN, sensitivity; SPE, specificity; PLR, positive likelihood ratio; NLR, negative likelihood ratio; DOR, diagnostic odds ratio; CI, confidence interval.

### Subgroup analysis

The results of the subgroup analysis are shown in Table 2. Of the 7 included trials, there were 4 Asian studies and 3 European studies. Both the pooled sensitivity (0.70 vs. 0.82) and specificity (0.90 vs. 0.93) were slightly lower for the European studies than for the Asian studies, but significant heterogeneity was observed in these subgroups.

### Sensitivity analysis and publication bias

A single study included in this meta-analysis was evaluated each time to determine the influence of the individual data set on the sensitivity and specificity. The results were not impacted by a single study (Table 2). The P-value for the Q test and the I^2^ value also showed that no single study affected the heterogeneity in this meta-analysis. These findings showed that the results of our study were relatively stable.

**Figure 7:**
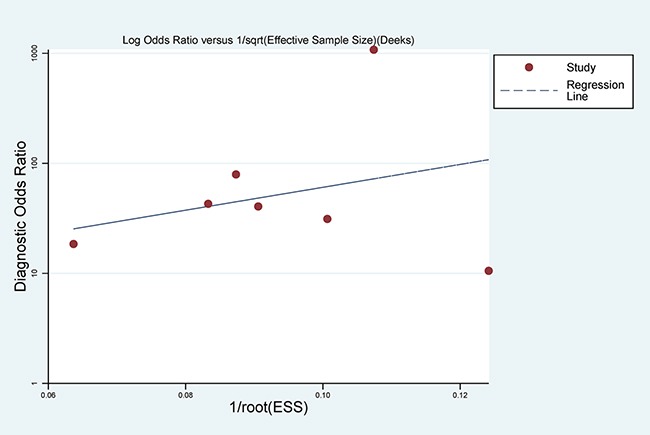
Deek’s Funnel Plot Asymmetry Test for the assessment of potential publication bias

With regard to publication bias, Deeks’ funnel plot asymmetry test was conducted. No significant publication bias was found in the pooled analysis of these studies (P = 0.431) (Figure 7).

## DISCUSSION

The aim of this study was to conduct a systematic review to evaluate the accuracy of urine HE4 as a biomarker for ovarian cancer, a disease with high mortality and a high rate of diagnosis at a late stage. The detection of biomarkers in urine is a less invasive and more convenient way of identifying ovarian cancer. There are some reasons why urinary HE4 analysis may be more sensitive than serum HE4 analysis in certain cases of ovarian cancer. The protein profile in urine may be less complex than that in blood, and certain proteins may be more stable in urine than in blood; moreover, a urinary test would be more convenient than a more invasive blood test [18]. In addition, antibodies to HE4 may be present in women with ovarian carcinoma or with certain types of infertility [19]. These antibodies can be retained by the kidneys; thus, the titre can be analysed at early stages with higher accuracy than serum HE4 measurements [20]. Several urine markers have been identified, including eosinophil-derived neurotoxin, a fragment of osteopontin [21], mesothelin [22, 23], and Bcl-2 [24].

In 2010, Hellstrom et al reported that measuring HE4 in urine can identify patients with ovarian cancer with a similar sensitivity to assaying sera. However, changes in urine creatinine and urine protein concentration are related; therefore, the ratio of urine HE4/creatinine in early and late ovarian cancer was a better predictor than urine HE4 alone. This result was consistent with the conclusions by Liao et al. However, Macuks et al.[12] found that the diagnostic value of urine HE4 alone was better than that of the urine HE4/creatinine ratio, and Jian HQ [13] reported that there was no significant difference between the two measurements. The predictive value of the urinary HE4/creatinine ratio is better than that of urine HE4, which requires further testing for validation.

To the best of our knowledge, this study is the first meta-analysis to evaluate urine HE4 as a tumour marker for ovarian cancer. The results of the meta-analysis indicated that the pooled sensitivity and specificity of urine HE4 were 0.76 (95% CI, 0.72–0.80) and 0.92 (95% CI, 0.89–0.94) respectively, meaning that 76% of the ovarian cancer patients had high HE4 levels and that 92% of the non-ovarian cancer patients had low HE4 levels. DOR combines the strengths of sensitivity and specificity as a prevalence-independent indicator and is useful from a statistical standpoint [25]. DOR values range from 0 to infinity, with higher values indicating better discriminatory test performance [26]. The DOR value of 37.90 indicates that urine HE4 could be a useful biomarker for ovarian cancer diagnostics. In addition, a good diagnostic test with the best performance will have a PLR greater than 10 and an NLR less than 0.1 [27]. Nevertheless, this review found an overall PLR of 8.39 and an NLR of 0.23, indicating that urine HE4 can neither confirm nor exclude patients with ovarian cancer. Overall, although the sensitivity was compromised, urine HE4 had a good specificity in the diagnosis of ovarian cancer. Urine HE4 was an effective biomarker for ovarian cancer diagnosis.

Of the 7 included trials, only 4 Asian studies had directly compared the diagnostic value of urine HE4 and serum HE4. The results of meta-analyses showed that compared with serum HE4, urine HE4 had demonstrated a higher pooled sensitivity (0.82 vs. 0.74), and a higher pooled specificity (0.93 vs. 0.88). When compared with serum HE4, the summary DOR of urine HE4 was better (56.40 vs. 19.39), the pooled PLR of urine HE4 was better (10.22 vs. 5.69), and the pooled NLR was higher (0.30 VS. 0.20). The AUC of SROC for urine HE4 and serum HE4 were 0.92 and 0.95, respectively, which indicated that urine HE4 may be superior to serum HE4 in screening ovarian cancer. More studies are needed for future analyses.

Heterogeneity is a potential problem when interpreting the results of any meta-analysis [28]. In the present study, we found considerable heterogeneity among the included studies, and the Spearman analysis showed that heterogeneity could not be explained by a threshold effect. Subgroup analyses were performed based on ethnicity to explore the potential source of heterogeneity. Urine HE4 exhibited higher diagnostic accuracy for ovarian cancer detection in non-Caucasian persons than in Caucasian persons, but the heterogeneity was still very high. We speculate that the inclusion of ovarian cancer patients at different disease stages and the use of different cut-off values, types of instruments, and reagent sources contributed to the heterogeneity. Due to the limited number of eligible studies, we could not further elucidate the source of the heterogeneity. Thus, these hypotheses need to be investigated in future studies.

In addition to heterogeneity, there were other limitations. First, we included studies that enrolled healthy women in the control group, which may have resulted in a false increase in the pooled specificity for differentiating benign disease. Second, our study evaluated the performance of HE4 regardless of menopausal status, age, smoking status, decreased renal function, or estimated glomerular filtration rate [29, 30]. This may have introduced bias in the conclusions, which may make it hard to extrapolate the results. Third, as the research focusing on the diagnosis value of urine HE4 has only been conducted recently, there are only seven studies and the sample sizes of studies included in this meta-analysis are small, which may appear small-study effects. Fourth, there was significant heterogeneity among our included studies, which might indicate the presence of factors that introduce bias. Fifth, only articles published in English or Chinese were included in this meta-analysis, which could have introduced inevitable bias.

In conclusion, the current evidence suggests that urine HE4 is a useful diagnostic biomarker for ovarian cancer, and diagnosis using this non-invasive method could be highly efficient. However, due to the quality of the included studies and the fact that patients suspected of having ovarian cancer were not included, additional studies must be performed to verify the results of this study and the accuracy of this method in terms of achieving high-quality diagnoses.

## MATERIALS AND METHODS

### Search strategy

The meta-analysis was conducted following the criteria of Preferred Reporting Items for Systematic Reviews and Meta-Analyses (PRISMA) [31]. The protocol of this review was registered in PROSPERO (http://www.crd.york.ac.uk/PROSPERO/), and the registration number is CRD42016038755. The protocol and the PRISMA checklist have been uploaded as supporting information (S1 PRISMA Checklist and S1 Protocol).

### Inclusion and exclusion criteria

Inclusion criteria were as follows: (1) a case group of patients with a diagnosis of pathologically confirmed primary ovarian cancer and a control group of women with pathologically confirmed benign gynaecological disease and/or healthy women; this criterion, pathological examination of biopsy specimens, represents the diagnostic gold standard in accordance with the International Federation of Gynecology and Obstetrics guidelines; (2) studies regarding the diagnostic potential of urine HE4 for ovarian cancer; and (3) studies reporting sufficient information regarding the sensitivity and specificity of HE4 to construct two × two contingency tables.

Exclusion criteria were as follows: (1) letters, editorials, case reports, reviews or studies without complete data; (2) sensitivity and specificity were not reported or could not be calculated; and (3) studies with duplicate data reported in other studies.

### Data extraction

Two reviewers independently selected all of the literature based on the inclusion and exclusion criteria. Then, these reviewers extracted the following information from the eligible studies: author, year of publication, country of origin, sample size, assay methods, cut-off values, and data regarding true positive (TP), false positive (FP), false negative (FN) and true negative (TN) rates with histology as the gold standard. Disagreements on the eligibility of studies were resolved by full-text review and discussion; if a consensus could not be reached, then a third reviewer was consulted.

### Methodological quality assessment of the included studies

Two reviewers independently assessed the quality and potential for bias of all the studies using the revised Quality Assessment of Diagnostic Accuracy Studies (QUADAS-2) tool [32]. This tool is composed of two parts: the risks of bias and concerns regarding applicability. The risks of bias were assessed in four domains: patient selection, index test, reference standard, and flow and timing. The concerns regarding applicability were assessed in three domains: patient selection, index test, and reference standard. A study received an overall judgement of “low risk of bias” or “low concern regarding applicability” if it was judged as “low” on all domains. In contrast, it would be judged as “risk of bias” or having “concerns regarding applicability” if it scored “high” or “unclear” in one or more domains. This process was performed using Review Manager 5 (http://ims.cochrane.org/revman/download).

### Statistical analyses

The statistical analysis was performed using Meta-DiSc software (version 1.4) [33]. We extracted the number of participants with a TP, FP, FN or TN from each study to calculate the pooled sensitivity, specificity, positive likelihood ratio (PLR), negative likelihood ratio (NLR), diagnostic odds ratio (DOR) and corresponding 95% confidence intervals (95% CI). Forest plots of accuracy indexes were also constructed. To describe the relationship between test sensitivity and specificity, a summary receiver operating characteristic (SROC) curve was constructed based on the TP and FP rates. An area under the curve (AUC) close to 1 indicated a good diagnostic performance of urine HE4 [34].

A threshold effect is an important cause of heterogeneity in diagnostic testing that can be confirmed by the Spearman correlation coefficient and probability (P) value between the logistic regression of sensitivity and 1 – specificity, and P < 0.05 indicated a significant threshold effect [35]. Heterogeneity caused by non-threshold effects was assessed by the Q and I^2^ tests. P < 0.10 for the Q test or an I^2^ value greater than 50% indicates substantial heterogeneity, and the random effects model was applied; otherwise, a fixed effects model was adopted. Subgroup and sensitivity analyses were performed to explore the potential sources of between-study heterogeneity. Deeks’ funnel plots were used to assess potential publication bias with STATA 12.0 software [36]. All statistical tests were two-sided, and P < 0.05 was considered to indicate statistical significance.
